# Critical Review on cathode–electrolyte Interphase Toward High-Voltage Cathodes for Li-Ion Batteries

**DOI:** 10.1007/s40820-022-00917-2

**Published:** 2022-08-16

**Authors:** Jijian Xu

**Affiliations:** Department of Chemical and Biomolecular Engineering, University of Maryland College Park, College Park, MD 20742 USA

**Keywords:** Cathode–electrolyte interphase, High-voltage cathodes, Interfacial chemistry, Electrolyte design, Batteries.

## Abstract

The thermal stability window of current commercial carbonate-based electrolytes is no longer sufficient to meet the ever-increasing cathode working voltage requirements of high energy density lithium-ion batteries. It is crucial to construct a robust cathode–electrolyte interphase (CEI) for high-voltage cathode electrodes to separate the electrolytes from the active cathode materials and thereby suppress the side reactions. Herein, this review presents a brief historic evolution of the mechanism of CEI formation and compositions, the state-of-art characterizations and modeling associated with CEI, and how to construct robust CEI from a practical electrolyte design perspective. The focus on electrolyte design is categorized into three parts: CEI-forming additives, anti-oxidation solvents, and lithium salts. Moreover, practical considerations for electrolyte design applications are proposed. This review will shed light on the future electrolyte design which enables aggressive high-voltage cathodes.

## Highlights

- A critical assessment of cathode–electrolyte interphase (CEI) for high-voltage cathode electrodes in Li-ion cells.
- Fundamental understanding of why interfacial interphase is important to electrochemical performance and further elaboration on how to design robust CEI interphase.
- Emerging theoretical simulations and advanced in situ characterizations helps to unveil the mystery of CEI are summarized.

## Graphical abstract

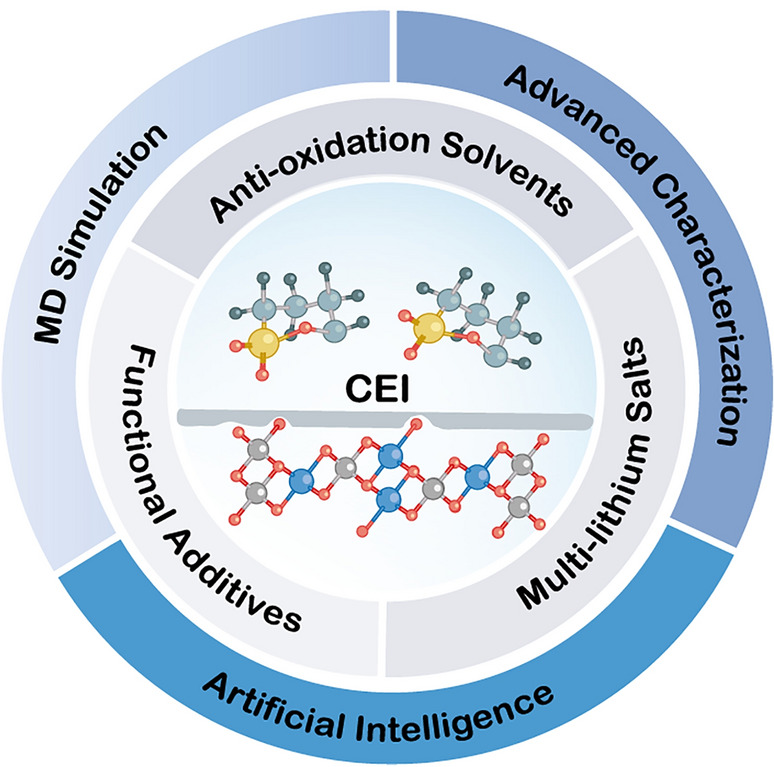

## Introduction and Scope

Along with the transition to a net-zero emissions future, there is a consistently growing demand for high energy density lithium-ion batteries with high voltage and high specific capacity [1, 2]. The simplest method to further improve the energy density of lithium-ion batteries is to increase the upper cutoff voltages. Taking the representative LiCoO_2_ as an example, the discharge capacity increases from 170 to 220 mAh g^−1^ by changing the upper cutoff voltages from 4.3 to 4.6 V [3]. However, creating a battery that can withstand high upper cutoff voltages while maintaining low side effects is no small feat. Cutoff voltage fluctuations accelerate interfacial reactions between the cathodes and electrolytes which will inevitably lead to serious consequences such as rapid capacity decay or even battery breakdown.

Since 2011, extensive works on cathode modification and electrolyte design have emerged in the hope of suppressing or even eliminating such interfacial reactions. There are multiple effective cathode modification strategies such as heteroatom doping and surface coating [4, 5]. Heteroatom doping was applied to stabilize the crystal structure of primary particles and inhibit the undesired electrode–electrolyte interfacial reactions [6–9]. Likewise, surface coating strategies including oxides, fluorides, and phosphates have been put forward to prevent electrolyte penetration and transition metal dissolution [10–14]. Atomic layer deposition and molecular layer deposition outperform various surface coating techniques, enabling controllable coating with atomic-level precision, excellent uniformity, and conformity [15, 16]. Conformal surface coating can be effective even under a high temperature of 55 °C: exhibiting capacity retention of 89.4% for Ni-rich cathode [17]. Another promising strategy aims to enhance electrolyte stability by modulating electrode/electrolyte interfacial reactions directly through electrolyte design. In the history of Li-ion batteries, the electrolyte-derived interphase on the anode was observed and defined as “solid electrolyte interphase (SEI),” which is a milestone [18]. In parallel, the interphase formed on the cathode is named “cathode–electrolyte interphase (CEI).” The role of CEI was once overlooked because there is no thermodynamic driving force for electrolyte oxidation for commercial batteries operating within 4.3 V [19]. The understanding of CEI becomes increasingly important due to the requirement of high voltage operation [20–23]. In situ formation of robust CEI via rational electrolyte design is the most promising strategy to separate the electrolyte from active cathodes and prolong the cycle life under high-voltage operation due to its ease of regulation by various components and self-healing ability. A review of CEI from the perspective of electrolyte design could provide fundamental guidance for further research.

This review aims to recount the history of CEI from its concept evolution to practice, including the cumulative cognition of CEI compositions and formation, the latest knowledge about CEI brought by advanced characterizations and modeling effects, and the design principles of CEI especially from the perspective of practical electrolyte design, and future research needs on this topic. All the electrolytes in our review are liquid unless noted; otherwise, the discussion of solid-state electrolytes is not included in the scope of this paper.

## CEI Chemistry in Evolution

Unlike SEI on the anode side, the important role of CEI was not realized until attempts were made to increase the cutoff voltage beyond the oxidation stability of electrolytes [24]. It is academically accepted that there is virtually no thermodynamic driving force for electrolyte oxidation on most conventional positive electrode materials. However, this law only applies to thermal stability windows below 4.3 V and fails when the cutoff voltage increases.

In addition, the view that CEI is not present on the cathode surface below 4.3 V was also found to be misleading. A lot of studies linked oxidation stability with the highest occupied molecular orbital (HOMO) energy [5]. In general, molecules with higher HOMO energy are more vulnerable to oxidation, from which researchers derive oxidation stability higher than the actual value, leading to the conclusion that CEI is nonexistent under 4.3 V. The new study, however, shows that the oxidation potential strongly depends on the local environment, meaning that there is no direct correlation between HOMO energies and experimentally observed oxidation stability [25, 26]. Therefore, it is more reasonable to use HOMO energy as a qualitative assessment of possible oxidation stability. In contrast, quantum chemistry (QC) calculation which takes the local solvents and anion environments into consideration is a promising direction for predicting oxidation stability [26].

Analogous to the case of graphite anodes, when high voltage operation exceeds the oxidation stability limits of organic electrolytes, a robust CEI is required to suppress side reactions. Before discussing how to design a powerful CEI, a comprehensive understanding of CEI is necessary, which is a challenge due to its sensitive chemical nature, complex formation process entangled with both electrolyte composition as well as surface chemistries of cathodes, and the lack of reliable characterization tools.

### Composition and Formation Mechanisms of CEI

**Fig. 1:**
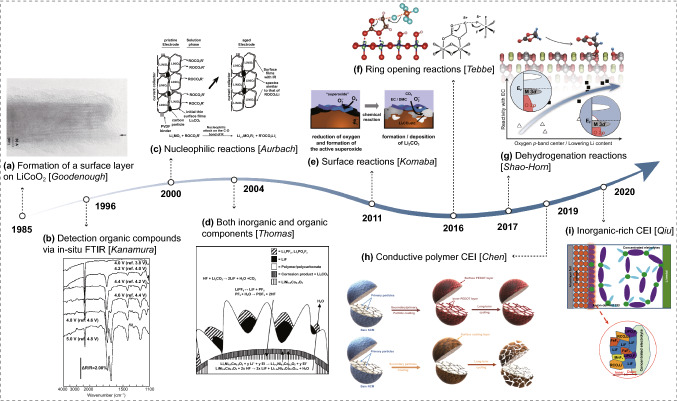
A brief historic evolution of CEI on positive electrodes. **a** Formation of a surface layer on LiCoO_2_ was first suggested by Goodenough et al. early in 1985. Copyright from Ref. [29]. **b** Kanamura et al. detected carboxylate species using in situ FTIR. Copyright from Ref. [30]. **c** Nucleophilic reactions mechanism was proposed by Aurbach for nucleophilic cathodes. Copyright from Ref. [28]. **d** The compositions of the CEI layer are further identified from XPS results. Copyright from Ref. [31]. **e** Surface reaction mechanism of the formation of Li_2_CO_3_. Copyright from Ref. [39]. **f** Ring-open reaction of ethylene carbonate. Copyright from Ref. [42]. **g** Dehydrogenation reaction on the cathode surface. Copyright from Ref. [44]. **h** Illustration of the structural stability of artificial conductive polymer CEI. Copyright from Ref. [32]. **i** Schematic of LiF-rich CEI formed in the concentrated electrolyte. Copyright from Ref. [33]

Pioneering works have been carried out to study the CEI even though the validity of the CEI concept was still doubted in the early 1990s [27, 28]. Selected studies show a brief historic evolution of the mechanism of CEI compositions and formation is presented in Fig. 1. A surface layer on LiCoO_2_ was firstly suggested by Goodenough et al. through the analysis of impedance spectra in conjunction with electron microscopy observation (Fig. 1a) [29]. The composition of the surface layer was later investigated using an in situ Fourier transform infrared (FTIR) spectroscopy, and Fig. 1b demonstrates the presence of carboxylate groups on the surface of cycled LiCoO_2_ thin film electrode [30]. A bi-layer CEI model consisting of an inner layer of polymer/polycarbonate and outer layer of LiF as well as precipitated species like Li_x_PO_y_F_z_, phosphorus oxides was proposed according to the X-ray photoelectron spectroscopy (XPS) analysis of cycled LiMn_2_O_4_ electrodes (Fig. 1d) [31]. Figure 1h illustrates that artificial CEI of conductive polymer on Ni-rich cathodes can effectively suppress the undesired layered to spinel/rock-salt phase transformation and enhance the capacity under high-voltage operation [32]. Meanwhile, a LiF-rich CEI formed in the concentrated electrolyte can also stabilize the cathode structure and improve the electrochemical performance of lithium-rich cathode (Fig. 1i) [33]. One vital but debatable issue in CEI chemistry is the role of fluorinated species, such as LiF. On the one hand, less LiF was reported to result in a thin CEI film with low impedance to enhance high-voltage performance [34, 35]. On the other hand, LiF-rich CEI layers have been well reported with superior cycling performance, especially with concentrated electrolytes [33, 36, 37]. With the development of environmental transmission electron microscopy (TEM), in situ visualization of LiF formation on CEI in LiPF_6_/propylene carbonate was achieved, which shows a remarkable self-healing ability of LiF [38]. Undoubtedly, this work is a milestone that deepens our understanding of LiF formation on CEI and guides us toward improving CEI chemistry.

It is extremely challenging to fully understand the CEI formation mechanism due to the complicated CEI compositions which are still not completely identified so far. However, a number of meaningful explorations have been conducted in recent years. Based on spectroscopic measurements and structural analysis, exchange reactions and nucleophilic reaction mechanisms were proposed for different cathode materials [28]. In the case of nucleophilic cathodes such as LiNiO_2_, the electrode is covered by ROCO_2_Li originating from direct reactions between the active materials and the electrolyte solutions (Fig. 1c). However, the existence of all CEI components cannot be explained by this mechanism alone, and more CEI formation mechanisms have been proposed later. As shown in Fig. 1e, Yabuuchi et al. raised a surface reaction mechanism where oxygen can be reduced to superoxide to attack carbonate solvents to form Li_2_CO_3_ [39]. It is worth mentioning that the cathode materials undergo surface reconstruction or reduction of transition metal oxidation state in contact with the electrolytes [40, 41], indicating the charge transfer between cathodes and electrolytes. CEI was found to be dominated by ethylene carbonate open-ring reaction activated by PF_5_ derived from LiPF_6_ decomposition (Fig. 1f) [42]. Other ethylene carbonate open-ring reactions initiated by electron-abstraction, proton-abstraction, and Lewis base were also discussed [43]. Shao-horn and co-workers found that the ethylene carbonate dissociation leads to hydroxylation of the cathode surface, namely the dehydrogenation reaction mechanism [44]. Importantly, the tendency of ethylene carbonate dissociation is strongly cathode material dependent. Proton transfer, also known as H-transfer reaction, between solvents on the cathode surface followed by solvent oxidation has recently been found to be universal [45]. The oxidation stability of common solvents including carbonates, sulfones, phosphates as well as ether significantly drops when coupled with H-transfer [46, 47]. Such a mechanism understanding shed light on manipulating the CEI chemistry by bringing specific components closer to the cathode surface to facilitate desired redox reactions.

### Interaction Between CEI and SEI

**Fig. 2:**
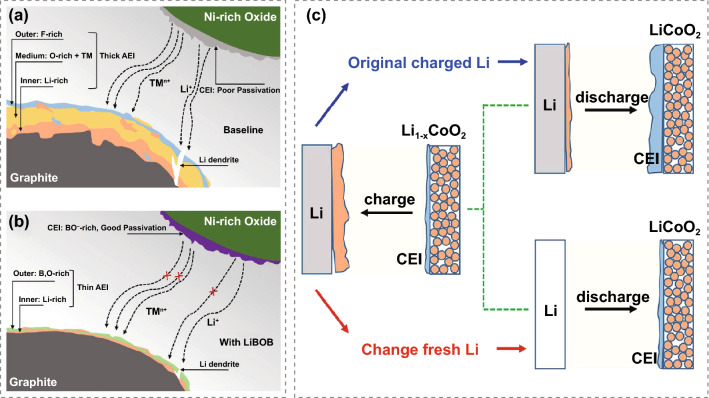
Schematic illustration of the interaction between CEI and SEI caused by transition metal crossover with **a** baseline electrolyte and **b** LiBOB-added electrolyte. Copyright from Ref. [48]. **c** Li anode in LiCoO_2_/Li battery after the first charge (4.6 V) was replaced by fresh Li to investigate the interaction between CEI and SEI. Copyright from Ref. [49]

CEI and SEI were usually studied independently as separate components on the cathode side and anode side. More attention should be paid to the correlation between the two, given that both are important components in the Li-ion battery system. Recently, it was revealed that the SEI transforms from a thick “three-layer” to a thin “two-layer” architecture by tuning the CEI surface chemistry via the amount of lithium bis-(oxalate)borate (LiBOB) additive, demonstrating obvious CEI and SEI interaction (Fig. 2a and b) [48]. The modified CEI layer is composed of B_x_O_y_ species with extreme robustness against electrochemical abuse which can effectively prevent the transition-metal crossover, benefiting the formation of a thin (O-enriched exterior layer and Li-dominating interior layer) SEI. Li et al. highlighted the strong interaction between CEI on high voltage LiCoO_2_ cathode and SEI by quantitative XPS analysis of CEI/SEI components and evolution [49]. The CEI components only slightly changed with fresh Li metal or graphite anode replaced at the charge state, while the CEI thickness increased rapidly with the original charged Li metal during the discharge process, as presented in Fig. 2c. In another example, the generated gas species at a cutoff potential above 4.2 V was migrated to, and then interacted with the SEI layer, as verified by gas chromatography-mass spectrometry measurement [50]. With the ongoing research efforts, increasing evidence has indicated the interaction between CEI and SEI [51, 52]. In parallel with Li-ion batteries, synergistically strengthening the SEI and CEI leads to ultra-stable cycle life of dual-ion batteries [53–55].

## Micro-Cognition of CEI via Novel Technologies

### Advanced Characterizations

**Fig. 3:**
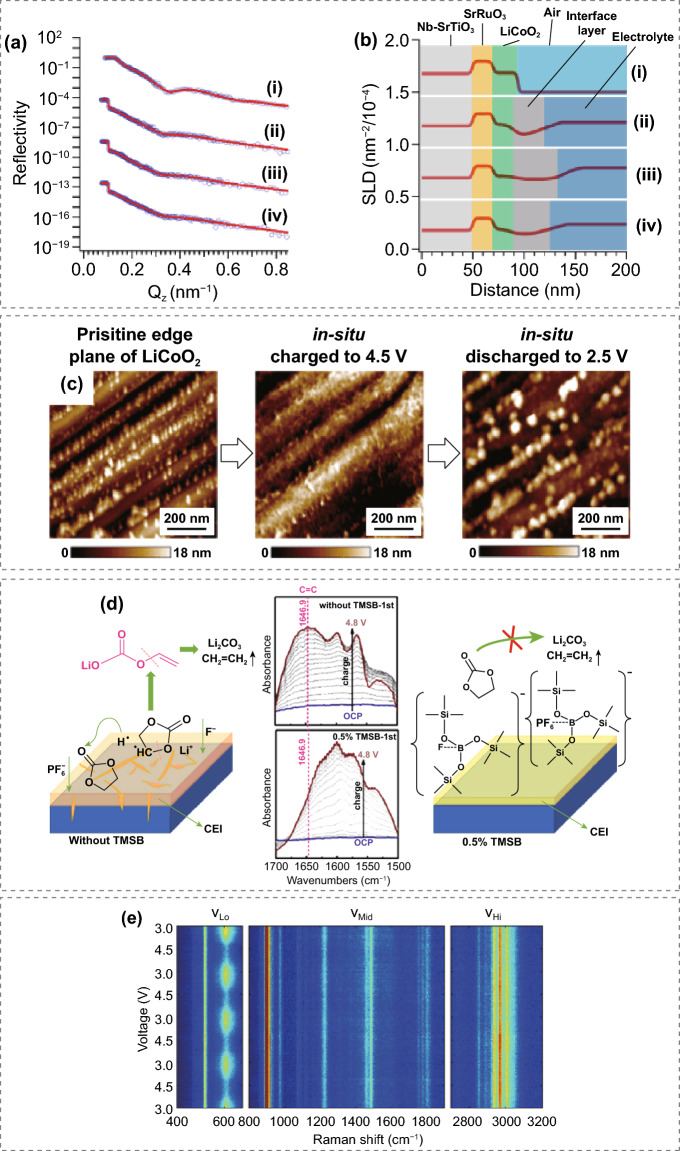
**a** Neutron reflectivity and **b** analyzed scattering length density for samples at different charge states. Copyright from Ref. [57]. **c** In situ AFM images of the CEI formation and decomposition on the edge of LiCoO_2_ crystal Copyright from Ref. [58]. **d** Operando ATR − FTIR spectra and schematic of CEI formation during the first charging process at the cathode surface in electrolytes with and without tris(trimethylsilyl)-borate additive Copyright from Ref. [59]. **e** Operando Raman spectral evolution acquired for a LiNi_0.33_Co_0.33_Mn_0.33_O_2_ electrode Copyright from Ref. [60]

CEI is dynamic during the charge/discharge cycling, and therefore, advanced operando characterizations are crucial to understanding the CEI evolution [56]. Changes in structure and composition of the CEI layer can be monitored by in situ neutron reflectometry [57]. As shown in Fig. 3a and b, the CEI thickness increased to 48.8 nm at 4.2 V for sample (iii) and decreased to 35.6 nm at 3.3 V for sample (iV), suggesting a growth/dissociation of the CEI layer during Li^+^ extraction/insertion. In situ atomic force microscopy (AFM) visualized the morphological changes of the CEI layer up to a high voltage of 4.5 V, revealing that the CEI films are only formed at the edge plane of LiCoO_2_ crystal and decomposed at the discharge state (Fig. 3c) [58]. An operando-attenuated total reflection—Fourier transform infrared (ATR—FTIR) technique was developed to study the dynamic mechanism of CEI formation in real time [59]. It was found that the addition of tris(trimethylsilyl)-borate additive can prevent the continuous decomposition of ethylene carbonate at high voltage and promote the stability of the CEI film. Raman bands of CEI exhibited substantial dynamics in strong correlation with the state-of-charge of the LiNi_0.33_Co_0.33_Mn_0.33_O_2_ electrode by a monolayer of deposited Au nanocubes [60]. Ideally, the in situ characterization techniques should bring minimum interruption to the operating cells and be under real operating conditions [61]. Learning from the anode side, in situ mass spectrometry and Cryo-TEM [62, 63], which dynamically investigate the SEI formation, can also be applied to monitor and visualize CEI formation. Emerging nanoscale X-ray tomography combined with artificial intelligence and machine learning might be able to develop predictive models to analysis the impact of CEI on cell performance [64, 65]. All these techniques have helped us understand the cell failure mechanism. More importantly, we should try to guide the optimization of better electrolytes based on observations from various characterizations.

### Molecular Dynamics (MD) Simulation and Machine Learning

**Fig. 4:**
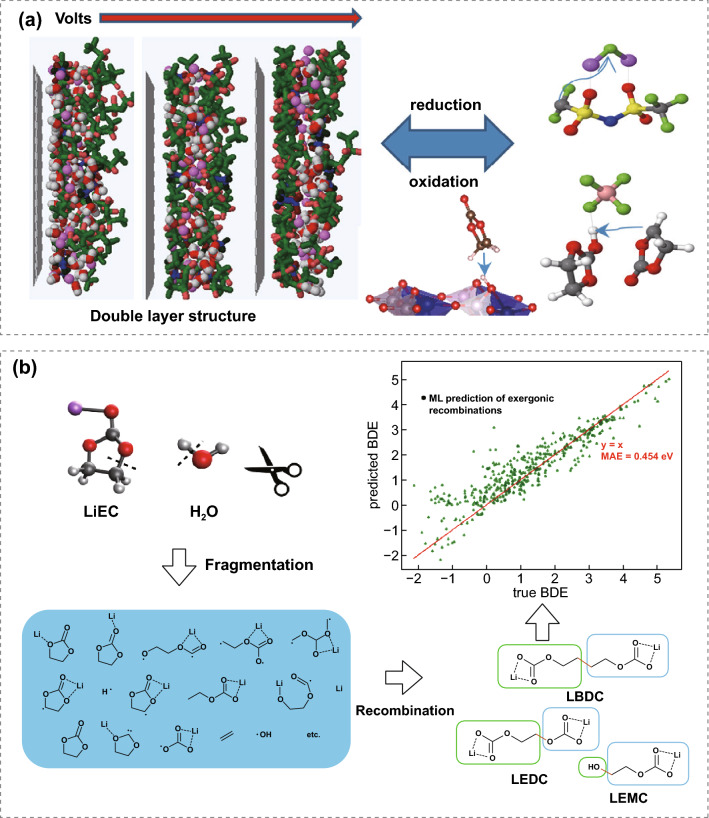
**a** Snapshots on the interfacial structure from MD simulations of (Dimethyl carbonate)_1.2_LiTFSI and preferential decomposition. Copyright from Ref. [45]. **b** Data-driven machine learning workflow for generating relevant molecules. Copyright from Ref. [70]

Nowadays, MD simulations play an important role in investigating electrolyte solvation structure, the formation of CEI and its evolution. MD simulation applied in batteries can trace back to the late 1990s [66, 67]. Oleg and co-works investigated the interfacial chemistry on the cathode side using classic MD simulation with applied electrode potentials [45, 68]. On the cathode electrode surface, highly concentrated electrolytes were found to exclude the solvent molecules away and selected anions could be preferentially absorbed for decomposition (Fig. 4a). Density functional theory (DFT) in combination with ab-initio molecular dynamics was conducted to understand the electrolyte role in CEI formation, showing that an electrolyte with high fluorine content can induce a robust fluorinated CEI [69]. Note that the box size and simulation time are very limited due to their high computation expense for ab-initio molecular dynamics simulations. The Kristin A. Persson group first incorporated machine learning to predict the decomposition pathway of electrolyte components [70, 71]. As shown in Fig. 4a–b, reaction network was developed to explore possible intermediates and reaction pathways, obtaining 570 candidate molecules and identifying two novel lithium ethylene monocarbonate formation mechanisms. Machine learning models trained upon the known properties including dielectric constant, HOMO energy, and etc. provide a way to rapidly screen new electrolyte solvents and even blended solvents with different ratios. Experimental trial-and-error testing of new compositions of electrolytes can be significantly accelerated with data-driven machine learning [72]. Such data-driven artificial intelligence continues to transform electrolyte design and shows great potential for further optimization of liquid electrolytes and Li-ion cells.

## Robust CEI: From Electrolyte Design

### CEI-Forming Additives

Adding functional additives that improve cathode stability is the most cost-effective strategy to make conventional carbonate-based electrolytes compatible with aggressive high-voltage cathodes. During the past decades, various types of functional additives and their combination have been investigated [73–75]. In this section, we mainly focus on the CEI-forming additives in carbonate-based electrolytes, and other functional additives, such as overcharge protectants and fire-retardant agents, are out of scope. However, related research could be found in other good reviews [76, 77].

**Fig. 5:**
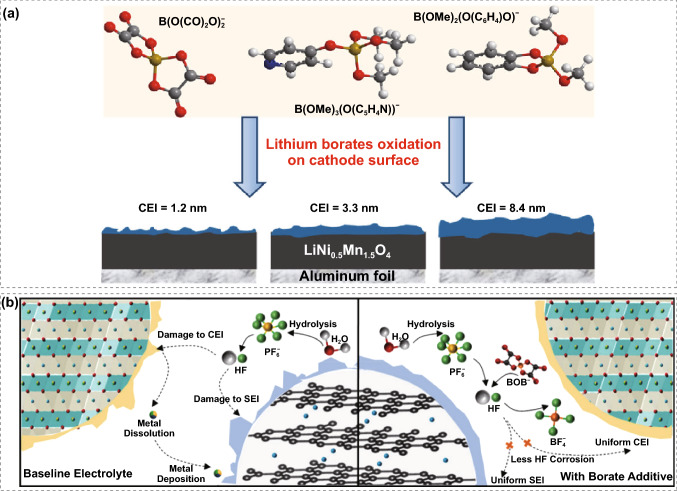
**a** Effect of lithium borate additives on CEI formation on LiNi_0.5_Mn_1.5_O_4_ electrodes. Copyright from Ref. [87]. **b** Schematic of enhanced full cell performance for LiBOB additive electrolyte in high-voltage system. Copyright from Ref. [88]

Based on whether the additives participate in interfacial reactions, additives can be classified as sacrificial and non-sacrificial. Sacrificial electrolyte additives electrochemically decompose before the host electrolytes and thereby form CEI on the cathode–electrolyte. These additives include unsaturated carbonate, boron-containing additives [78], nitrogen-containing additives [79, 80], fluorine-containing additives [81], silicon-containing additives [82], phosphorus-containing additives [83], and sulfur-containing additives [84]. Among them, boron-containing chemicals, such as LiBOB, tris (trimethylsilyl) borate [85], trimethyl borate [86], and triethyl borate, were particularly effective. This is because additives containing electron-deficient boron atoms could coordinate with anion PF_6_^−^, which lowers the oxidation potential of the baseline electrolyte and participates in the formation of the protective CEI. A systematic comparison of the CEI formation on LiNi_0.5_Mn_1.5_O_4_ cathodes with three different lithium borate electrolyte additives has been conducted [87]. As shown in Fig. 5a, the CEI layer thickness increases in the order of lithium catechol dimethyl borate > lithium 4-pyridyl trimethyl borate > LiBOB, suggesting a strong correlation between the CEI layer thickness and reactivity of the additive. A mechanism study for the LiBOB-enabled 4.5 V lithium-rich layered oxides||graphite full cells was further conducted (Fig. 5b), confirming the formation of a uniform interphase with B-F species on high-voltage cathodes under cryo-condition [88]. In situ formation of F- and B-rich CEI layer on LiNiO_2_ cathode was demonstrated using LiDFOB as an additive, maintaining high capacity retention of > 80% (400 cycles) at a high cutoff voltage of 4.4 V [89].

Another requirement of being sacrificial additives is that they must possess a lower reduction potential than the solvent; otherwise, it needs to create a protective SEI on the anode surface at the same time. With this in line, many studies have developed blended additives or multifunctional additives by combining the advantages of different additives. Synergistic effects of dual additives on protecting the cathodes under high potentials have been investigated. A blend of SEI film-forming additive (vinylene carbonate) and CEI film-forming additive (1,3-propane sultone) resulted in improved capacity retention [90]. Dual additives (trimethyl borate + fluoroethylene carbonate and trimethyl borate + tetramethylene sulfone) in the commercial electrolyte can lower the oxidation potential and form a thinner, more stable CEI, enabling the performance of LiNi_0.8_Co_0.1_Mn_0.1_O_2_ cathodes charged to 4.5 V [91]. The combination of tris (trimethylsilyl) phosphite and lithium difluoro(oxalato)borate in the electrolyte not only forms a robust CEI but also improves the thermal stability. (Capacity retains more than 91% when stored at 60 °C for 50 days.) [92] A ternary electrolyte additive system consisting of 2% prop-1-ene-1, 3-sultone + 1% methylene methane disulfonate + 1% tris-(trimethylsilyl)-phosphite significantly improves the capacity retention of LiNi_0.4_Mn_0.4_Co_0.2_O_2_||graphite pouch cells cycled at constant current up to 4.5 V [93]. In addition, several ternary blends of triphenylphosphate with different film-forming additives were reported to have better high-voltage performance compared to electrolytes with a single additive [94]. Alternatively, integrating the nitrile and borate groups into a single additive (tris(2-cyanoethyl) borate) results in the in situ-formed rich N and B CEI layer and thus enabling single-crystal Ni-rich cathode to operate at cutoff voltage as high as 4.7 V with outstanding cycling stability [95]. Following the same logic, tris-(trimethylsilyl)-phosphite was used as a multifunctional additive, in which the trivalent phosphorus can scavenge oxygen gas in the cell, the electrophilic silicon can remove nucleophilic lithium oxide species, and the silyl ether component can prevent transition metal dissolution [96, 97].

Non-sacrificial electrolyte additives are emerging very recently, which activate the CEI formation without self-sacrificing. As well known, sacrificial additives will gradually consume through the interfacial reactions, hence deteriorating the cycling stability of lithium-ion cells eventually. Recent research revealed that employing non-sacrificial electrolyte additives can perfectly address the aforementioned concern. Methyl diphenylphosphonate as a non-sacrificial additive was evaluated by Zhang et al. [98], which can stabilize the LiNi_0.8_Mn_0.1_Co_0.1_O_2_ cathode/electrolyte interface by physical absorption. The first principle calculation demonstrates that the methyl diphenylphosphonate additive forms a stable pyramid structure with Mn ions and Li ions on the cathode surface, which also contributes to the thermal stability of the cells at high temperatures. As reported by Wang et al., the local fluoroethylene carbonate (FEC)-Li^+^ configuration triggers the oxidative decomposition of the otherwise inert FSI^−^, while the FEC additive remains stable against the electrochemical cycling [99].

Overall, electrolyte additives have been proven to be effective in enhancing the electrochemical performance of high-voltage Li-ion cells by forming stable CEI. Considering the abundance of the additive species and their numerous combinations, there is still plenty of space in taking full advantage of the commercial carbonate-based electrolytes.

### Anti-oxidation Solvents

In comparison with traditional commercial carbonate solvents, anti-oxidation solvents not only possess greater stability under high voltage, but also contribute to a robust CEI interface when involved in interfacial reactions. In commercial lithium-ion cell systems, carbonate solvents are the most popular ones, exhibiting good performance at cutoff voltages below 4.3 V. However, as the voltage increases, their thermodynamic limitations manifest themselves, which, coupled with enhanced cathode surface reactions, lead to extensive deterioration in cell performance. By introducing anti-oxidation solvents with greater stability such as fluorinated solvents, sulfones, nitrile, and ionic liquid, the electrochemical performance of Li-ion cells with high-voltage cathodes can be effectively improved.

**Fig. 6:**
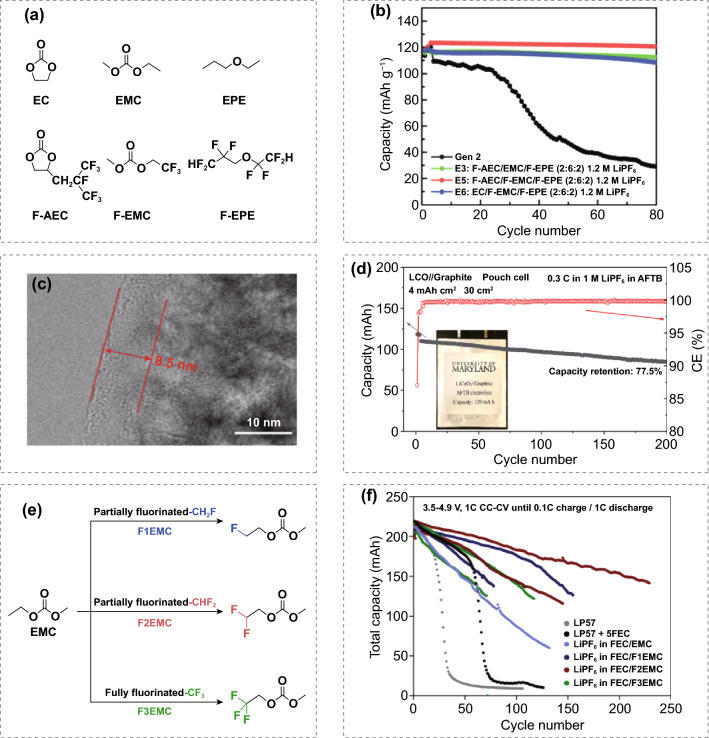
**a** Chemical structure of the baseline carbonate, ether fluorinated counterparts. **b** Cycling capacity retention of Li_4_Ti_5_O_12_/LiNi_0.5_Mn_1.5_O_4_ cells with baseline electrolyte and fluorinated electrolytes at 55 °C. Copyright from Ref. [100]. **c** TEM images of cycled LiCoO_2_ electrode and **d** cycle performance of the single-crystalline LiCoO_2_//graphite pouch cell in the fluorinated electrolyte. Copyright from Ref. [3]. **e** Molecular structures of fluorinated ethyl methyl carbonate solvents. **f** Cycling behavior of LiNi_0.5_Mn_1.5_O_4_//graphite pouch cells using different electrolytes with 1 M main salt and FEC/fluorinated-EMC = 3/7 volume ratio at 1C charge/discharge. LP57: 1 M LiPF_6_ in ethylene carbonate/ethyl methyl carbonate (EC/EMC, 3/7 by volume). LP57 + 5FEC: LP57 + 5% FEC (by weight). Copyright from Ref. [104]

Among the various anti-oxidation solvents, fluorinated solvents have been well investigated in the hope of extending the electrochemical stability window of electrolytes. The fluorine substitution in the solvents shows improvement in oxidation stability due to the strong electron-withdrawing effect of fluorine atoms and also contributes to the enrichment of CEI with fluorinated species. Amine’s group demonstrated that a fluorinated carbonate solvent-based electrolyte provides superior voltage stability on the 5.0 V spinel LiNi_0.5_Mn_1.5_O_4_ cathode at both ambient and elevated temperature at 55 °C (Fig. 6a–b) [100]. Note that fluorine substitution results in simultaneously higher oxidation stability and higher reduction potential. Considering higher reduction potential may lead to instability on the anode side [101], a stable Li_4_Ti_5_O_12_ anode was employed to accurately evaluate the beneficial effect on the cathode side. Wang and co-workers reported an all-fluorinated electrolyte can form a highly fluorinated, conformal, and dense CEI consisting of inorganic species with a thickness of 5–10 nm that stabilizes not only the high voltage LiNi_0.8_Mn_0.1_Co_0.1_O_2_ (efficiency ~ 99.93%) and LiCoPO_4_ (efficiency ~ 99.81%) cathodes, but also lithium metal (plating/stripping, ~ 99.2%) [102]. As a result, full cells retain ~ 93% of their original capacities after 1,000 cycles at a practical loading of 2.0 mAh cm^−2^. By forming a robust CEI layer (~ 8.5 nm) in the all-fluorinated electrolyte, 4.5 V high loading (4 mAh cm^−2^) LiCoO_2_//graphite pouch cell delivered excellent capacity retention of 80% after 500 cycles (Fig. 6c-d) [3]. However, such an all-fluorinated electrolyte consisting of ultra-high extent of fluorinated solvents might be an “over-kill” [103]. A family of fluorinated ethyl methyl carbonates with different numbers of F atoms was systematically studied to reveal the effects of fluorination extent of carbonate solvents on battery performance (Fig. 6e-f). Yu et al. found that fully fluorinated solvents are not necessarily desirable. Instead, the degree of fluorination needs to be rationally tuned in order to optimize the Li-ion cell performance [104]. Knowing exactly which component functions as the key fluorinating agent for the CEI interphase constitutes the key knowledge to enabling future battery chemistries.

Sulfone-based electrolytes offer another pathway toward enabling aggressive high-voltage cathodes. Angell first reported a sulfone-based electrolyte of 1 M LiPF_6_ dissolved in ethyl methoxyethyl sulfone, showing remarkable anodic stability of above 5.0 V versus lithium [105]. Later, the electrochemical stability of five sulfone-based electrolytes was evaluated using lithium bis (trifluoromethanesulfonyl) imide (LiTFSI) as a lithium salt. Among these, ethyl methyl sulfone or tetramethyl sulfone exhibited the highest anodic stability [106]. Significant anodic stability is achieved at the cathode–electrolyte interface because of the sulfone group in the molecular which helps to lower the HOMO level. The compatibility between the sulfones and graphite anodes could be addressed by various approaches. Amine and co-workers synthesized β-fluorinated sulfone, which is not only resistant to oxidation on the high voltage LiNi_0.6_Mn_0.2_Co_0.2_O_2_ cathode but also reductively stable toward the graphite anode [107]. Moreover, β-fluorinated sulfone is a non-flammable solvent with reduced lithium solvating power, mitigating the transition metal dissolution of the cathodes. Ultimately, β-fluorinated sulfone-based electrolytes enable the stable long-term cycling of graphite/LiNi_0.6_Mn_0.2_Co_0.2_O_2_ full cells with the highest capacity retention of 81% after 400 cycles. Together with concentrated lithium bis (fluorosulfonyl) imide (LiFSI) derived SEI, a sulfone-based electrolyte enables a high voltage (4.85 V) graphite/LiNi_0.5_Mn_1.5_O_4_ full cell to operate over 1000 cycles, retaining 70% of its first-cycle discharge capacity. QC calculations predict that the decomposition of sulfone results in polymerizable products, leading to a thin, sulfur-based CEI which are corroborated by XPS and cryogenic-transmission electron microscopy [108]. Considering sulfones as the electrolyte solvent, one annoying drawback that should be pointed out is the high melting point. Mixing sulfones with other solvents is the most effective method to tackle the issue. By incorporating fluoroethylene carbonate into tetramethylene sulfone, the mixed electrolyte forms ultra-thin fluorine and sulfur-rich CEI layer [109].

Nitrile-based electrolytes with a wide electrochemical window also serve as attractive candidates for high-voltage Li-ion cells. Generally, the anti-oxidation feature of nitriles is believed to be caused by the highly nucleophilic -CN groups which can be preferentially chemisorbed on the surface of high-voltage cathodes, generating a monolayer and preventing their oxidative decomposition [79, 110]. However, it is unlikely that a chemisorbed monolayer could be electrochemically tough enough to resist the thermodynamic driving force of electrolyte decomposition. Taking succinonitrile as an example, Li et al. found the succinonitrile-derived N-containing CEI interphase also makes an important role in improving high-voltage stability [111]. DFT calculation revealed the interaction between salt anion and succinonitrile solvent greatly reduces its resistance against oxidation, thus making the formation of N-containing CEI possible. On the other hand, Cui’s team demonstrated that the succinonitrile solvent in succinonitrile-based deep eutectic electrolyte reacts with the charged LiCoO_2_ cathode by using in situ XRD and in situ FTIR techniques [112]. A uniform, N-containing CEI layer was also observed on the LiNi_0.5_Co_0.2_Mn_0.3_O_2_ for electrolyte with succinonitrile and fluoroethylene carbonate simultaneously as solvents [113]. Concentrated nitrile electrolyte consisting of a solvent mixture of succinonitrile and acetonitrile exhibits interfacial stability at a high cutoff voltage of 4.9 V due to the formation of uniform CEI layers [114].

### Lithium Salts

The conventional wisdom believes that lithium salts remain stable during electrochemical cycling, especially for LiPF_6_ which dominates the commercial lithium salts market nowadays. Johansson first explored the intrinsic anion stability of lithium salts by electronic structure calculations [115]. Limited understanding has been reported toward anion-derived CEI in nonaqueous electrolytes. In advancing to more aggressive cathode chemistry at higher voltages, new lithium salts that can contribute to stable CEI formation are urgently needed.

Two representative examples are LiBOB and lithium difluorooxalatoborate (LiDFOB), both of which can decompose and in situ form CEI at high cutoff voltages. LiBOB was reported as anodic unstable at voltages higher than 4.2 V [116], in turn, can be used to form stable CEI. The CEI formed in LiBOB electrolyte enables LiNi_0.8_Co_0.15_Al_0.05_O_2_ cathode with better rate capability when compared to the LiPF_6_ counterpart [117]. LiBOB was also applied to other high-voltage cathodes (LiNi_0.5_Mn_1.5_O_4_, LiCoPO_4_) with remarkably improved capacity retention and decreased impedance [118, 119]. Ex situ surface analysis via FTIR and XPS of the cycled LiNi_0.5_Mn_1.5_O_4_ cathodes suggests the addition of LiBOB leads to a thinner CEI film containing oxalate species. Unfortunately, LiBOB has limited solubilities in carbonate solvents, restricting its applications.

**Fig. 7:**
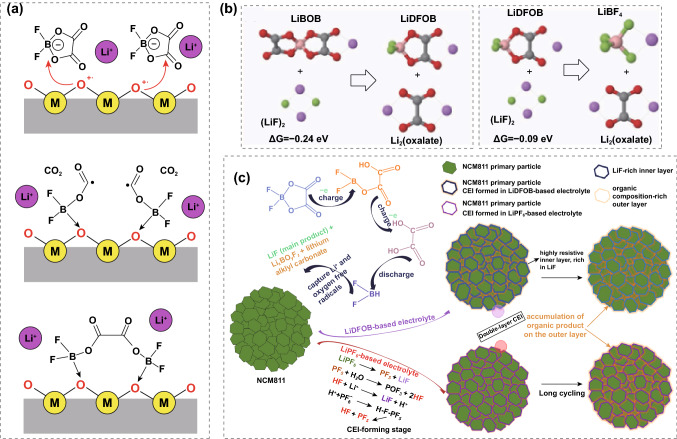
**a** Schematic illustration of the possible reaction path of the CEI formation on the cathode electrode surface using LiDFOB as a lithium salt. Copyright from Ref. [121]. **b** Free energy ∆G for LiF + LiBOB and LiF + LiDFOB reactions. Copyright from Ref. [122]. **c** Scheme of hierarchical CEI formation process in LiDFOB-based electrolyte and the LiPF_6_ counterpart. Copyright from Ref. [123]

LiDFOB was found to possess the combined merits of its parent salts of LiBOB and LiBF_4_ [120]. The role of LiBOB and LiDFOB on CEI was investigated via electron paramagnetic resonance spectroscopy and both were found involved in one-electron oxidation with the elimination of CO_2_ and the generation of an acyl radical (Fig. 7a) [121]. Acyl radicals anchored to bridging oxygens on the cathode surfaces can form dimers through cross-recombination and pile up to form a coating on the cathode surfaces. Differently, another mechanism of the reaction between LiBOB/LiDFOB and dissociated F^−^ anion for forming CEI was proposed based on QC calculations (Fig. 7b) [122]. The BOB^−^ reaction with F^−^ was found to be more energetically favorable, which is supported by the XPS results with a strong signal ascribed to B-F bonds. Although the specific reaction mechanism remains to be further explored, experimentally, there are already many reports with much improved high-voltage performance with the adoption of LiDFOB salt. As illustrated in Fig. 7c, a bi-layer CEI consisting of LiF-rich inner layer and Li_x_BO_y_F_z_-rich outer layer is in situ constructed on the LiNi_0.8_Mn_0.1_Co_0.1_O_2_ cathode through the oxidative decomposition of LiDFOB [123]. Such a robust CEI effectively protects the cathode from reacting with electrolyte, thereby boosting the capacity retention of 69.8% after 400 cycles as well as a high specific capacity of 127.5 mAh g^−1^ at 10C.

Single lithium salt still has a tough road ahead in accomplish massive challenges of high-voltage Li-ion cells. Naturally, the synergistic effect between lithium salts and solvents, or dual-salt has great potential to improve the overall electrochemical performance [124, 125]. A combination of LiDFOB salt and sulfur-containing solvents of ethylene sulfite, dimethyl sulfite, and sulfolane has been investigated for 5 V high voltage cells [126]. Among these, the LiDFOB-sulfolane-derived CEI films are suggested to be denser and more stable. Similar synergistic action between LiDFOB salt and sulfolane solvent was also reported on LiNi_0.8_Co_0.15_Al_0.05_O_2_ cathode chemistry with more LiF formed in the CEI layer, which improves the cycle performance of the cell [127]. Recently, a dual-salt electrolyte (LiDFOB/LiBF_4_ in carbonate solvents) enabled the best performance for anode-free pouch cell-80% capacity retention after 90 cycles [128, 129]. The LiDFOB and LiBF_4_ lithium salts were continuously consumed during cycling at 4.3 V, forcing a limited cycle life. Moreover, a dual-salt electrolyte (2 M LiTFSI + 2 M LiDFOB in dimethoxyethane, DME) allowed stable cycling of LiNi_1/3_Mn_1/3_Co_1/3_O_2_ cathode at 4.3 V despite the limited oxidative stability of DME (< 4 V) [130]. The key to breaking the voltage limitation for ether-based electrolytes is the formation of stable interfacial layers on cathodes. Four imide-borate dual-salt electrolytes in carbonate solvent were investigated, showing the electrochemical stability in the order of LiTFSI-LiBOB > LiTFSI-LiDFOB > LiFSI-LiDFOB > LiFSI-LiBOB [131]. It is also worth mentioning that LiDFOB was shown to be the best inhibitor of Al corrosion in LiFSI-based dual-salt electrolytes [132]. In the search for an ideal lithium salt, LiFSI was considered a magic salt due to its unique ability to dissociate and form protective SEIs [18, 133, 134]. However, the corrosion of aluminum current collectors is a longstanding barrier for imide salts including LiFSI and LiTFSI [135].

**Fig. 8:**
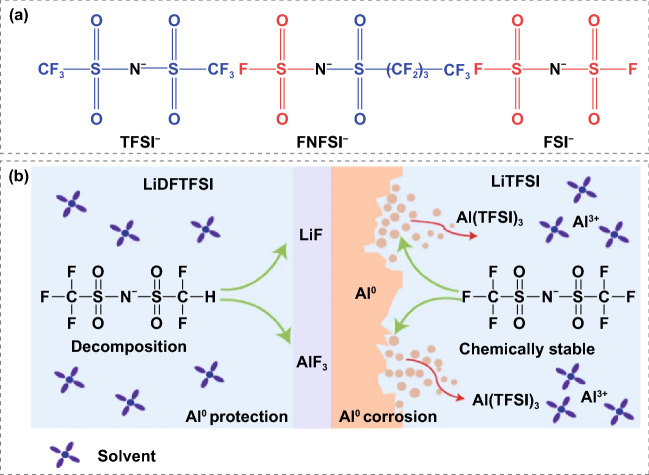
New lithium salt design: **a** Structures of TFSI^–^, FNFSI^–^, and FSI^–^ anions. Copyright from Ref. [140]. **b** Structures of DFTFSI– and TFSI– anions and schematic illustration of the possible Al corrosion. Copyright from Ref. [141]

While progress has been made to alleviate Al corrosion by blending with other lithium salts [136–138], new imide-based lithium salts offer a radical solution that can intrinsically address the corrosion issues through rational molecular design. A novel lithium salt, lithium (fluorosulfonyl) (nonafluorobutanesulfonyl) imide (LiFNFSI), which does not corrode aluminum was developed by replacing the -CF_3_ group with longer perfluorinated alkyl chains [139]. The inorganic fluorosulfonyl (FSO_2_-) group in LiFNFSI was suggested to be beneficial for forming a protective layer on Al surface to suppress its corrosion, see molecular structure in Fig. 8a [140]. Likewise, another non-corrosive sulfonimide salt, lithium (difluoromethanesulfonyl) (trifluoromethanesulfonyl) imide (LiDFTFSI) that critically prevent the anodic dissolution of the aluminum current collector at high voltages of at least 4.2 V versus Li/Li^+^, was reported recently [141]. The unstable nature of Al(DFTFIS)_3_ in carbonate solvents makes it easy to decompose to form AlF_3_ and LiF protective layers, thus preventing further anodic dissolution (Fig. 8b). Additionally, the LiDFTFSI also enables the formation of an excellent CEI layer on the LiNi_1/3_Mn_1/3_Co_1/3_O_2_ cathode.

It is difficult to synthesize new lithium salts, and most of the newly synthesized lithium salts are not directly usable as sole main salt. These new lithium salts enrich our toolbox for manipulating electrolytes and may yield surprising results when coupled with anti-oxidation solvents or/and CEI-forming additives.

Regarding lithium salts, another parameter that must be mentioned is the concentration. The “1 molarity (M) legacy” of conventional electrolytes stemmed from the quest of maximum ionic conductivities [142]. Notwithstanding, deviation from this "ideal" fixation has opened a new direction known as “solvent-in-salt” electrolytes or “super-concentrated” electrolytes [143–145]. Past MD simulations of concentrated electrolytes have shown that there is a large portion of TFSI^–^ anions in the inner-Helmholtz layer at the cathode side, compelling the solvent away and preventing it from oxidizing [146]. In a high concentration regime, one may expect CEI generated from the decomposition of the anions. As reported, the oxidation stability of concentrated 10 M LiFSI in carbonate electrolyte can be mainly ascribed to the anion-derived fluorine-rich CEI [147]. The F-rich CEI successfully stabilizes the LiNi_0.6_Mn_0.2_Co_0.2_O_2_ cathode at a high cutoff voltage of 4.6 V, showing remarkable capacity retention of 86% after 100 cycles. As well, the CEI layer formed in concentrated 3 M LiPF_6_ in carbonate electrolyte was found to be highly homogeneous and robust, which not only effectively inhibits the dissolution of transition metals but also stabilizes the cobalt-free cathode structure [33]. In sharp comparison, Fe and Mn elements are detected on cycled cathodes in 1 M electrolyte because of the uneven and fragile organic-rich CEI layer. A more conformal, anion-based CEI of up to 4.4 V can be obtained using a combination of LiFSI and LiTFSI at a higher concentration in DME in anode-free LiNi_0.6_Mn_0.2_Co_0.2_O_2_ cell configurations [148]. QC calculations anticipated that all sulfolane molecules are coordinated by Li^+^ in the high-concentration electrolyte which slows the decomposition of sulfolane and leads to polymerized CEI [108]. Despite the success of concentrated electrolytes in stabilizing the high-voltage cathodes, high concentration itself induces compromises in conductivity and viscosity. There is a continuous trend to change the salt concentration back to 1 M or even low concentration while maintaining the merits of high concentration [149, 150]. A group of localized high-concentration electrolytes has been extensively developed to build protective interphases onto both the anode and the high-voltage cathodes [151–153].

### Practical Considerations

In addition to the fundamental understanding of electrolyte design, we need to pay attention to the critical requirements including cost, eco-friendly, safety, and wide temperature range operation for practical applications. Cost is always the primary factor in commercialization. Electrolytes account for around 5%–15% in the battery cost [154, 155]. Undoubtedly, the adoption of new lithium salts, solvents, or additives will drive the cost of electrolytes up. The good news is that the prices of Li-ion batteries have fallen by 97% since their commercialization in the late 1990s, in particular, about 38% of the cost reduction is caused by the increased battery charge density [155]. It means that advanced electrolytes enable high-voltage cathodes to increase energy density and thereby reduce overall cost at the cell level. Nevertheless, the importance of cost cannot be overemphasized, and we should always keep cost in mind during electrolyte design. Furthermore, all the electrolyte components should be eco-friendly, exhibiting the lowest environmental impact.

Upon increasing the energy density by high-voltage cathodes, battery safety becomes more critical [20]. Thermodynamically, higher operation voltage corresponds to a higher risk of electrolyte decomposition, gas generation, and therefore a higher likelihood of safety hazards. To make safer batteries, flammable carbonate-based electrolytes can be replaced by non-flammable electrolytes [156]. It is well acknowledged that introducing flame-retardant solvents in electrolytes results in non-flammability [157, 158], but more rigorous abuse tests, such as nail penetration or heating, are required [159, 160]. A deeper understanding of the thermal runaway mechanism and the design principles of electrolytes for safer batteries would be highly desired.

The widespread application of batteries calls for a correspondingly wide operating temperature range. Traditionally, efforts have focused on thermal management strategies, but Li-ion battery is inherently related to the electrolyte, SEI, and CEI layers [161, 162]. At low temperatures, down to −20 °C, liquid electrolytes confront freezing issues and resultant sluggish ion transportation through the SEI/CEI layers. Low-temperature operation requires electrolytes with low freezing points and low resistance SEI/CEI layers. At high temperatures, up to 60 °C, LiPF_6_ salt begins to decompose together with the volatility of the organic solvents and severe transition metal dissolution [163]. High-temperature operation requires electrolytes with high thermal stability and inorganic-rich interphases with low solubility. Some innovative works including liquified gas electrolytes and all-fluorinated solvents have been demonstrated to enable impressive cycling performance on the low-temperature side [164–166]. However, most of them cannot work at high temperatures due to the low boiling point of solvents utilized. Therefore, how to design an electrolyte that enables Li-ion cells to operate within a wide temperature range (−30 ~  + 60 °C) remains a big challenge as well as an exciting opportunity. For specific electric vehicle applications, in addition to a wide temperature range, more stringent parameters such as calendar life (10 years), cycle life (1000 cycles), and cost ($100/kWh) are required [167].

## Summary and Perspective

The formation of a stable CEI is critical to achieving high voltage lithium-ion cells with long cycling life. Ideally, a CEI should be conformal to separate the electrolytes from cathode materials and self-healing to accommodate the non-uniform electrochemical reactions. Great progress has been made on the CEI components, morphology, and formation mechanism using operando characterizations together with MD simulations. Multiple strategies have been developed to construct robust CEI through electrolyte design including solvents featuring anti-oxidation, multi-lithium salts with synergy effects, as well as additives both sacrificial and non-sacrificial. However, the fundamental question of how to design a controllable CEI with tunable components, thickness, ion conductivity, etc., is still not fully answered. Given that electrolytes, CEI, and cathode materials dynamically interact with each other upon cycling, it is important to consider the following aspects: (i) Universal principles in constructing stable CEI. Emerging techniques such as data-driven analysis and artificial intelligence have shown great potential in the high-throughput screening of electrolytes, which might be able to establish a correlation between CEI and electrolyte composition; (ii) the exact transport mechanism of Li-ion across the CEI. Potentially, isotopic tracing combined with cryo-TEM can be smartly designed to dynamically track the Li-ion transportation crossing the CEI. More collaborations are required to gain insights into the roles of different electrolyte components on CEI formation, making further electrolyte optimization possible. By rational designing electrolytes, robust CEI can be constructed so that Li-ion cells with a long lifespan are achievable even under high voltage operation.
